# Supplementary material for: Visual recognition of social signals by a tectothalamic neural circuit
Source: Nature. 2022 Jul 13;608(7921):146–52. doi: 10.1038/s41586-022-04925-5 (PMC9352588; doi:10.1038/s41586-022-04925-5)
Supplement: Supplementary file 1 — Reporting Summary [file 41586_2022_4925_MOESM1_ESM.pdf]

nature portfolio

Corresponding author(s): Herwig Baier, Johannes Larsch

Last updated by author(s): May 6, 2022

Reporting Summary

Nature Portfolio wishes to improve the reproducibility of the work that we publish. This form provides structure for consistency and transparency in reporting. For further information on Nature Portfolio policies, see our Editorial Policies and the Editorial Policy Checklist.

Statistics

For all statistical analyses, confirm that the following items are present in the figure legend, table legend, main text, or Methods section.

n/a	Confirmed	
☐	☒	The exact sample size (*n*) for each experimental group/condition, given as a discrete number and unit of measurement
☐	☒	A statement on whether measurements were taken from distinct samples or whether the same sample was measured repeatedly
☐	☒	The statistical test(s) used AND whether they are one- or two-sided<br>*Only common tests should be described solely by name; describe more complex techniques in the Methods section.*
☒	☐	A description of all covariates tested
☐	☒	A description of any assumptions or corrections, such as tests of normality and adjustment for multiple comparisons
☐	☒	A full description of the statistical parameters including central tendency (e.g. means) or other basic estimates (e.g. regression coefficient) AND variation (e.g. standard deviation) or associated estimates of uncertainty (e.g. confidence intervals)
☐	☒	For null hypothesis testing, the test statistic (e.g. *F*, *t*, *r*) with confidence intervals, effect sizes, degrees of freedom and *P* value noted<br>*Give P values as exact values whenever suitable.*
☒	☐	For Bayesian analysis, information on the choice of priors and Markov chain Monte Carlo settings
☒	☐	For hierarchical and complex designs, identification of the appropriate level for tests and full reporting of outcomes
☐	☒	Estimates of effect sizes (e.g. Cohen's *d*, Pearson's *r*), indicating how they were calculated

Our web collection on statistics for biologists contains articles on many of the points above.

Software and code

Policy information about availability of computer code

Data collection	Bonsai 2.4.1<br>Leica LAS X 3.5.7<br>ScanImage 5.6
Data analysis	Python 3.9 with NumPy 1.21.0, Scipy 1.7.0, MatplotLib 3.4.2, Pandas 1.3.0, and additional packages. (Full python environments are available with our custom scripts on bitbucket).<br>Ants 1.9<br>Suite2p 0.9.3<br>ImageJ 1.53c

For manuscripts utilizing custom algorithms or software that are central to the research but not yet described in published literature, software must be made available to editors and reviewers. We strongly encourage code deposition in a community repository (e.g. GitHub). See the Nature Portfolio guidelines for submitting code & software for further information.

Data

Policy information about availability of data

All manuscripts must include a data availability statement. This statement should provide the following information, where applicable:

- Accession codes, unique identifiers, or web links for publicly available datasets
- A description of any restrictions on data availability
- For clinical datasets or third party data, please ensure that the statement adheres to our policy

All data to evaluate the conclusions in the paper and to reproduce the analysis are present in the paper or made publicly available. Raw HCR data, 2-photon time

series for individual neurons and behavior tracking data are available on Edmond: https://doi.org/10.17617/3.2QCFQP. The EM stack will be publicly available with a companion paper (Svara et al.).

Field-specific reporting

Please select the one below that is the best fit for your research. If you are not sure, read the appropriate sections before making your selection.

☒ Life sciences ☐ Behavioural & social sciences ☐ Ecological, evolutionary & environmental sciences

For a reference copy of the document with all sections, see nature.com/documents/nr-reporting-summary-flat.pdf

Life sciences study design

All studies must disclose on these points even when the disclosure is negative.

Sample size	The sample size was not pre-computed. We chose sample sizes typical in the field, expecting large effect sizes (greater than 1SD) and refrained from strong conclusions based on the absence of significance, which would be limited by modest sample size.
Data exclusions	No individual data points were excluded from the analysis. Animals damaged during the embedding, during imaging, or during ablation were not analyzed further as described in the methods section.
Replication	All findings described in the manuscript were analyzed and found to be consistent across multiple animals, except the EM stack, which only exists for N=1 animal. Animal numbers are indicated in each figure legend.
Randomization	For c-fos mapping, animals were randomly assigned to stimulus groups. Test and control groups for MTZ treatment were assigned randomly.
Blinding	The experimenters were not blinded to experimental conditions or genotype of the animals. We applied a standardized, fully automated analysis to data from all animals.

Reporting for specific materials, systems and methods

We require information from authors about some types of materials, experimental systems and methods used in many studies. Here, indicate whether each material, system or method listed is relevant to your study. If you are not sure if a list item applies to your research, read the appropriate section before selecting a response.

Materials & experimental systems

n/a	Involved in the study	
☒	☐	Antibodies
☒	☐	Eukaryotic cell lines
☒	☐	Palaeontology and archaeology
☐	☒	Animals and other organisms
☒	☐	Human research participants
☒	☐	Clinical data
☒	☐	Dual use research of concern

Methods

n/a	Involved in the study	
☒	☐	ChIP-seq
☒	☐	Flow cytometry
☒	☐	MRI-based neuroimaging

Animals and other organisms

Policy information about studies involving animals; ARRIVE guidelines recommended for reporting animal research

Laboratory animals	Zebrafish (Danio rerio), age: 7-22 days. Transgenic lines are specified in the methods section. The sex of zebrafish is likely determined but not yet differentiated at this stage and was thus not a factor for choosing experimental animals.
Wild animals	No wild animals were used in this study.
Field-collected samples	No field collected samples were used in this study.
Ethics oversight	All animal experiments were performed under the regulations of the Max Planck Society and the regional government of Upper Bavaria (Regierung von Oberbayern), approved protocols: ROB-55.2Vet-2532.Vet 03-15-16, ROB-55.2Vet-2532.Vet 02-16-31, and ROB55.2Vet-2532.Vet 02-16-122.

Note that full information on the approval of the study protocol must also be provided in the manuscript.
